# Supplementary material for: The Efficacy of Fluvoxamine in Anxiety Disorders and Obsessive-Compulsive Disorder: An Overview of Systematic Reviews and Meta-Analyses
Source: Pharmaceuticals (Basel). 2025 Feb 28;18(3):353. doi: 10.3390/ph18030353 (PMC11944676; doi:10.3390/ph18030353)
Supplement: Supplementary file 1 [file pharmaceuticals-18-00353-s001.zip › pharmaceuticals-3373851-supplementary.pdf]

THE EFFICACY OF FLUVOXAMINE IN ANXIETY DISORDERS AND OBSESSIVE-COMPULSIVE DISORDER: AN OVERVIEW OF SYSTEMATIC REVIEWS AND META-ANALYSES

SUPPLEMENTARY MATERIAL

Michel Haddad, Luiz Henrique Junqueira Dieckmann, Thiago Wendt Viola, Naielly Rodrigues da Silva, Jair de Jesus Mari

I. Supplementary material Section S1—Summary of heterogeneity findings of studies that reported this analysis

Author and year	Summary of findings
Hedges 2007	Not significant
Boyer 1995	Not significant
Williams 2020	Heterogeneity was substantial for the continuous outcome of symptom severity (i.e. tau2 = 290.8; I2 = 93.8%) and the binary outcome response rate (i.e. tau2 = 0.1640; I2 = 62.1%). Heterogeneity estimates for the full network meta-analysis
Andrisano 2012	Heterogeneity was significantly observed in the full meta-analysis
Guaiana 2023	Analysis suggested a potential concern about heterogeneity in the comparison between fluvoxamine and placebo (OR 0.61, 95% PI 0.26 to 1.52)
Hansen 2008	Not significant
Liu 2018	Not significant
Du 2021	Findings not reported
Piccinelli 1995	Not significant
Ackerman 2002	Considerable heterogeneity was found within placebo-controlled comparisons of each drug in the full meta-analysis
Greist 1995	Findings not reported

II. Supplementary material Section S2—Assessment of methodological quality of the included systematic reviews (AMSTAR-2 application)

STUDY	1	2	3	4	5	6	7	8	9	10	11	12	13	14	15	16	Total	Qualitative score
Andrisano 2012	1	0	1	1	1	1	1	1	0	1	1	0	0	1	1	1	12	Moderate
Boyer 1995	1	0	1	1	0	0	0	1	0	0	1	0	0	0	0	0	5	Low
Du 2021	1	0	1	1	1	1	1	1	1	0	1	0	1	1	0	1	12	Moderate
Guaiana 2023	1	1	1	1	1	1	1	1	1	1	1	1	1	1	1	1	16	High
Hansen 2008	1	0	1	1	1	1	1	1	1	0	1	1	1	1	1	1	14	High
Liu 2018	1	1	1	1	1	1	1	1	1	1	1	1	1	1	1	1	16	High
Mochcovitch 2010	1	0	1	0	0	0	0	1	0	0	0	0	0	0	0	1	4	Low
Perna 2011	1	0	1	1	0	0	1	1	0	0	0	0	0	0	0	1	6	Low
Williams 2020	1	1	1	1	1	1	1	1	1	1	1	1	1	1	1	1	16	High
Piccinelli 1995	1	0	1	0	0	0	1	1	0	0	1	0	0	0	0	1	6	Low
Ackerman 2002	1	0	1	1	0	0	0	1	0	0	1	0	0	1	0	1	7	Low
Choi 2009	1	0	1	1	0	0	0	1	1	0	0	0	1	0	0	1	7	Low
Greist 1995	1	0	1	0	0	0	0	1	0	0	1	0	0	1	0	1	6	Low
Soomro 2008	1	0	1	1	1	1	1	1	1	0	1	1	1	1	1	1	14	High

Items and questions

1. Did the research questions and inclusion criteria for the review include the components of PIC
2. Did the report of the review contain an explicit statement that the review methods were established prior to the conduct of the review and did the report justify any significant deviations from the protocol?
3. Did the review authors explain their selection of the study designs for inclusion in the review?
4. Did the review authors use a comprehensive literature search strategy?
5. Did the review authors perform study selection in duplicate?
6. Did the review authors perform data extraction in duplicate?
7. Did the review authors provide a list of excluded studies and justify the exclusions?
8. Did the review authors describe the included studies in adequate detail?
9. Did the review authors use a satisfactory technique for assessing the risk of bias in individual studies that were included in the review?
10. Did the review authors report on the sources of funding for the studies included in the review?
11. If meta-analysis was performed did the review authors use appropriate methods for statistical combination of results?
12. If meta-analysis was performed, did the review authors assess the potential impact of RoB in individual studies on the results of the meta-analysis or other evidence synthesis?
13. Did the review authors account for risk of bias in individual studies when interpreting/ discussing the results of the review?
14. Did the review authors provide a satisfactory explanation for, and discussion of, any heterogeneity observed in the results of the review?
15. If they performed quantitative synthesis did the review authors carry out an adequate investigation of publication bias (small study bias) and discuss its likely impact on the results of the review?

16. Did the review authors report any potential sources of conflict of interest, including any funding they received for conducting the review?

III. Supplementary material Section S3—List of Included Randomized Controlled Trials

Randomized controlled trials included in Table 1. Characteristics of Included Systematic Reviews and Meta-Analyses on the Efficacy of Fluvoxamine in the Treatment of OCD. (n = 16)
Brar J, Sidana A, Chauhan N, Bajaj MK. Early Improvement as a Predictor of Treatment Response in Patients With Obsessive-Compulsive Disorder: A 12-Week Randomized Trial of Sertraline and Fluvoxamine. Prim Care Companion CNS Disord. 2022 Mar 31;24(2):21m03065.
Freeman CP, Trimble MR, Deakin JF, Stokes TM, Ashford JJ. Fluvoxamine versus clomipramine in the treatment of obsessive compulsive disorder: a multicenter, randomized, double-blind, parallel group comparison. J Clin Psychiatry. 1994 Jul;55(7):301-5.
Goodman WK, Kozak MJ, Liebowitz M, White KL. Treatment of obsessive-compulsive disorder with fluvoxamine: a multicentre, double-blind, placebo-controlled trial. Int Clin Psychopharmacol. 1996 Mar;11(1):21-9.
Goodman WK, Price LH, Rasmussen SA, Delgado PL, Heninger GR, Charney DS. Efficacy of fluvoxamine in obsessive-compulsive disorder. A double-blind comparison with placebo. Arch Gen Psychiatry. 1989 Jan;46(1):36-44.
Goodman, W, Greist, J, et al. Results of a double blind placebo controlled trial of a new serotoin reuptake inhibitor, sertraline, in the treatment of obsessive compulsive disorder. Psychopharmacology Bulletin, (26) 279-284
Greist JH. Fluvoxamine in OCD: a multicentre parallel design double-blind placebo controlled trial. Presented at the 18th Collegium Internationale NeuroPsychopharmacologicum Congress; June 29, 1992; Nice, France.
Hollander E, Koran LM, Goodman WK, Greist JH, Ninan PT, Yang H, Li D, Barbato LM. A double-blind, placebo-controlled study of the efficacy and safety of controlled-release fluvoxamine in patients with obsessive-compulsive disorder. J Clin Psychiatry. 2003 Jun;64(6):640-7.
Jenike MA, Baer L, Summergrad P, Weilburg JB, Holland A, Seymour R. Obsessive-compulsive disorder: a double-blind, placebo-controlled trial of clomipramine in 27 patients. Am J Psychiatry. 1989 Oct;146(10):1328-30.
Koran LM, McElroy SL, Davidson JR, Rasmussen SA, Hollander E, Jenike MA. Fluvoxamine versus clomipramine for obsessive-compulsive disorder: a double-blind comparison. J Clin Psychopharmacol. 1996 Apr;16(2):121-9.
Mallya GK, White K, Waternaux C, et al. Short- and long-term treatment of obsessive-compulsive disorder with fluvoxamine. Ann Clin Psychiatry 1992;4:77–80.
Milanfranchi A, Ravagli S, Lensi P, Marazziti D, Cassano GB. A double-blind study of fluvoxamine and clomipramine in the treatment of obsessive-compulsive disorder. Int Clin Psychopharmacol. 1997 May;12(3):131-6.
Mundo E, Rouillon F, Figuera ML, Stigler M. Fluvoxamine in obsessive-compulsive disorder: similar efficacy but superior tolerability in comparison with clomipramine. Hum Psychopharmacol. 2001 Aug;16(6):461-468.
Nakajima T, Kudo Y, Yamashita I. Clinical usefulness of Fluvoxamine Maleate (SME3110), a selective serotonin reuptake inhibitor, in the treatment of obsessive compulsive disorder: A dopuble blind, placebo-controlled study investigating the therapeutic dose range and the eEicacy of SME3110. Journal of clinical therapeutics & medicine (Rinshou Iyaku) 1996;12(3):409-37.
O'Connor KP, Aardema F, Robillard S, Guay S, Pélissier MC, Todorov C, Borgeat F, Leblanc V, Grenier S, Doucet P. Cognitive behaviour therapy and medication in the treatment of obsessive-compulsive disorder. Acta Psychiatr Scand. 2006 May;113(5):408-19.
Rasmussen SA, Eisen JL. The epidemiology and differential diagnosis of obsessive compulsive disorder. J Clin Psychiatry. 1992 Apr;53 Suppl:4-10.
Smeraldi, E. Erzegovesi, S, Bianchi, I, et al. Fluvoxamine v. Clomipramine treatment in obsessive compulsive disorder:a preliminary study. New Trends in Experimental and Clinical Psychiatry. 1992 (8) 63-65.

Randomized controlled trials included in Table 2. Characteristics of included systematic reviews and meta-analyses on the efficacy of fluvoxamine in the treatment of social anxiety disorder. (n = 6)
Asakura S, Tajima O, Koyama T. Fluvoxamine treatment of generalized social anxiety disorder in Japan: a randomized double-blind, placebo-controlled study. Int J Neuropsychopharmacol. 2007 Apr;10(2):263-74.
Davidson J, Yaryura-Tobias J, DuPont R, Stallings L, Barbato LM, van der Hoop RG, Li D. Fluvoxamine-controlled release formulation for the treatment of generalized social anxiety disorder. J Clin Psychopharmacol. 2004 Apr;24(2):118-25.
Stein DJ, Westenberg HG, Yang H, Li D, Barbato LM. Fluvoxamine CR in the long-term treatment of social anxiety disorder: the 12- to 24-week extension phase of a multicentre, randomized, placebo-controlled trial. Int J Neuropsychopharmacol. 2003 Dec;6(4):317-23.
Stein MB, Fyer AJ, Davidson JR, Pollack MH, Wiita B. Fluvoxamine treatment of social phobia (social anxiety disorder): a double-blind, placebo-controlled study. Am J Psychiatry. 1999 May;156(5):756-60.
van Vliet IM, den Boer JA, Westenberg HG. Psychopharmacological treatment of social phobia; a double blind placebo controlled study with fluvoxamine. Psychopharmacology (Berl). 1994 Jun;115(1-2):128-34.
Westenberg HG, Stein DJ, Yang H, Li D, Barbato LM. A double-blind placebo-controlled study of controlled release fluvoxamine for the treatment of generalized social anxiety disorder. J Clin Psychopharmacol. 2004 Feb;24(1):49-55.

Randomized controlled trials included in Table 3. Characteristics of included systematic reviews with meta-analyses on the efficacy of fluvoxamine in the treatment of panic disorder. (n = 15)
Asnis GM, Hameedi FA, Goddard AW, Potkin SG, Black D, Jameel M, Desagani K, Woods SW. Fluvoxamine in the treatment of panic disorder: a multi-center, double-blind, placebo-controlled study in outpatients. Psychiatry Res. 2001 Aug 5;103(1):1-14.
Bakish D, Filteau MJ, Charbonneau Y, Fraser G, West DL, Hooper CL, A double-blind, placebo-controlled trial comparing fluvoxamine and imipramine in the tratment of panic disorder with or without agarophobia. Presented at the CINP Regional Workshop, "Current Therapeutical Approaches in Panic and Other Anxiety Disorders", 20-22 Nov. 1993, Monte Carlo
Black D.W, Monahan P, Wesner R, Gabel J, Bowers W. The effect of fluvoxamine, cognitive therapy, and placebo on abnormal personality traits in 44 patients with panic disorder. J. Pers. Disord. 1996. 10, 185–194.
Black DW, Wesner R, Bowers W, Gabel J. A comparison of fluvoxamine, cognitive therapy, and placebo in the treatment of panic disorder. Arch Gen Psychiatry. 1993 Jan;50(1):44-50.
Den Boer JA, Westenberg HG. Effect of a serotonin and noradrenaline uptake inhibitor in panic disorder; a double-blind comparative study with fluvoxamine and maprotiline. Int Clin Psychopharmacol. 1988 Jan;3(1):59-74.
Den Boer JA, Westenberg HG. Serotonin function in panic disorder: a double blind placebo controlled study with fluvoxamine and ritanserin. Psychopharmacology (Berl). 1990;102(1):85-94.
Hoehn-Saric R, McLeod DR, Hipsley PA. Effect of fluvoxamine on panic disorder. J Clin Psychopharmacol. 1993 Oct;13(5):321-6.
Nair NP, Bakish D, Saxena B, Amin M, Schwartz G, West TE. Comparison of fluvoxamine, imipramine, and placebo in the treatment of outpatients with panic disorder. Anxiety. 1996;2(4):192-8.
Palatnik A, Frolov K, Fux M, Benjamin J. Double-blind, controlled, crossover trial of inositol versus fluvoxamine for the treatment of panic disorder. J Clin Psychopharmacol. 2001 Jun;21(3):335-9.
Perna G, Bertani A, Caldirola D, Gabriele A, Cocchi S, Bellodi L. Antipanic drug modulation of 35% CO_2 hyperreactivity and short-term treatment outcome. J Clin Psychopharmacol. 2002 Jun;22(3):300-8.

Pols HJ, Hauzer RC, Meijer JA, Verburg K, Griez EJ. Fluvoxamine attenuates panic induced by 35% CO2 challenge. J Clin Psychiatry. 1996 Nov;57(11):539-42.
Sandmann J, Lörch B, Bandelow B, Härtter S, Winter P, Hiemke C, Benkert O. Fluvoxamine or placebo in the treatment of panic disorder and relationship to blood concentrations of fluvoxamine. Pharmacopsychiatry. 1998 Jul;31(4):117-21.
Sharp DM, Power KG, Simpson RJ, Swanson V, Moodie E, Anstee JA, et al. Fluvoxamine, placebo and cognitive behaviour therapy used alone and in combination in the treatment of panic disorder and agoraphobia. Journal of Anxiety Disorders 1996;10(4):219-42.
van Vliet IM, den Boer JA, Westenberg HG, Slaap BR. A double-blind comparative study of brofaromine and fluvoxamine in outpatients with panic disorder. J Clin Psychopharmacol. 1996 Aug;16(4):299-306.
Westernberg HGM, den Doar JA, Selective monoamine uptake inhibitors and a serotonin antagonist in the treatment of panic disorder. Psychopharmacology Bulletin, 1989b (25), 119-123

IV. Supplementary material Section S4—Prisma Checklist

Section and Topic	Item #	Checklist item	Location where item is reported
**TITLE**			
Title	1	Identify the report as a systematic review.	Page 1
**ABSTRACT**			
Abstract	2	See the PRISMA 2020 for Abstracts checklist.	Page 1
**INTRODUCTION**			
Rationale	3	Describe the rationale for the review in the context of existing knowledge.	Page 2
Objectives	4	Provide an explicit statement of the objective(s) or question(s) the review addresses.	Page 2
**METHODS**			
Eligibility criteria	5	Specify the inclusion and exclusion criteria for the review and how studies were grouped for the syntheses.	Page 18
Information sources	6	Specify all databases, registers, websites, organisations, reference lists and other sources searched or consulted to identify studies. Specify the date when each source was last searched or consulted.	Page 18
Search strategy	7	Present the full search strategies for all databases, registers and websites, including any filters and limits used.	Page 18
Selection process	8	Specify the methods used to decide whether a study met the inclusion criteria of the review, including how many reviewers screened each record and each report retrieved, whether they worked independently, and if applicable, details of automation tools used in the process.	Page 19
Data collection process	9	Specify the methods used to collect data from reports, including how many reviewers collected data from each report, whether they worked independently, any processes for obtaining or confirming data from study investigators, and if applicable, details of automation tools used in the process.	Page 19
Data items	10a	List and define all outcomes for which data were sought. Specify whether all results that were compatible with each outcome domain in each study were sought (e.g. for all measures, time points, analyses), and if not, the methods used to decide which results to collect.	Page 19
	10b	List and define all other variables for which data were sought (e.g. participant and intervention characteristics, funding sources). Describe any assumptions made about any missing or unclear information.	Page 19
Study risk of bias assessment	11	Specify the methods used to assess risk of bias in the included studies, including details of the tool(s) used, how many reviewers assessed each study and whether they worked independently, and if applicable, details of automation tools used in the process.	Page 19
Effect measures	12	Specify for each outcome the effect measure(s) (e.g. risk ratio, mean difference) used in the synthesis or presentation of results.	Page 19
Synthesis methods	13a	Describe the processes used to decide which studies were eligible for each synthesis (e.g. tabulating the study intervention characteristics and comparing against the planned groups for each synthesis (item #5)).	Page 19
	13b	Describe any methods required to prepare the data for presentation or synthesis, such as handling of missing summary statistics, or data conversions.	Page 19
	13c	Describe any methods used to tabulate or visually display results of individual studies and syntheses.	Page 19
	13d	Describe any methods used to synthesize results and provide a rationale for the choice(s). If meta-analysis was performed, describe the model(s), method(s) to identify the presence and extent of statistical heterogeneity, and software package(s) used.	Page 19
	13e	Describe any methods used to explore possible causes of heterogeneity among study results (e.g. subgroup analysis, meta-regression).	Page 19
	13f	Describe any sensitivity analyses conducted to assess robustness of the synthesized results.	Page 19
Reporting bias assessment	14	Describe any methods used to assess risk of bias due to missing results in a synthesis (arising from reporting biases).	Page 19
Certainty	15	Describe any methods used to assess certainty (or confidence) in the body of evidence for an outcome.	Page 19

Section and Topic	Item #	Checklist item	Location where item is reported
assessment			
**RESULTS**			
Study selection	16a	Describe the results of the search and selection process, from the number of records identified in the search to the number of studies included in the review, ideally using a flow diagram.	Page 2
	16b	Cite studies that might appear to meet the inclusion criteria, but which were excluded, and explain why they were excluded.	-
Study characteristics	17	Cite each included study and present its characteristics.	Tables 1-3
Risk of bias in studies	18	Present assessments of risk of bias for each included study.	Supplementary Material
Results of individual studies	19	For all outcomes, present, for each study: (a) summary statistics for each group (where appropriate) and (b) an effect estimate and its precision (e.g. confidence/credible interval), ideally using structured tables or plots.	Pages 4-15
Results of syntheses	20a	For each synthesis, briefly summarise the characteristics and risk of bias among contributing studies.	Supplementary Material
	20b	Present results of all statistical syntheses conducted. If meta-analysis was done, present for each the summary estimate and its precision (e.g. confidence/credible interval) and measures of statistical heterogeneity. If comparing groups, describe the direction of the effect.	-
	20c	Present results of all investigations of possible causes of heterogeneity among study results.	Supplementary Material
	20d	Present results of all sensitivity analyses conducted to assess the robustness of the synthesized results.	-
Reporting biases	21	Present assessments of risk of bias due to missing results (arising from reporting biases) for each synthesis assessed.	-
Certainty of evidence	22	Present assessments of certainty (or confidence) in the body of evidence for each outcome assessed.	Tables 1-3
**DISCUSSION**			
Discussion	23a	Provide a general interpretation of the results in the context of other evidence.	Page 16
	23b	Discuss any limitations of the evidence included in the review.	Page 17
	23c	Discuss any limitations of the review processes used.	Page 17
	23d	Discuss implications of the results for practice, policy, and future research.	Pages 17-18
**OTHER INFORMATION**			
Registration and protocol	24a	Provide registration information for the review, including register name and registration number, or state that the review was not registered.	Page 20
	24b	Indicate where the review protocol can be accessed, or state that a protocol was not prepared.	Page 20
	24c	Describe and explain any amendments to information provided at registration or in the protocol.	-
Support	25	Describe sources of financial or non-financial support for the review, and the role of the funders or sponsors in the review.	Page 20
Competing interests	26	Declare any competing interests of review authors.	Page 20
Availability of data, code and other materials	27	Report which of the following are publicly available and where they can be found: template data collection forms; data extracted from included studies; data used for all analyses; analytic code; any other materials used in the review.	-

From: Page MJ, McKenzie JE, Bossuyt PM, Boutron I, Hoffmann TC, Mulrow CD, et al. The PRISMA 2020 statement: an updated guideline for reporting systematic reviews. BMJ 2021;372:n71. doi: 10.1136/bmj.n71.
This work is licensed under CC BY 4.0. To view a copy of this license, visit https://creativecommons.org/licenses/by/4.0/
